# The transcription factors regulating intervertebral disc development

**DOI:** 10.1002/jsp2.1081

**Published:** 2020-02-20

**Authors:** Ryo Nakamichi, Hiroshi Asahara

**Affiliations:** ^1^ Department of Molecular and Experimental Medicine The Scripps Research Institute La Jolla California; ^2^ Department of Orthopaedic Surgery Okayama University Graduate School of Medicine, Dentistry, and Pharmaceutical Sciences Okayama Japan; ^3^ Department of Systems Biomedicine Tokyo Medical and Dental University Tokyo Japan

**Keywords:** annuls fibrosus, intervertebral disc, mesenchymal stem cells, nucleus pulposus, transcription factor

## Abstract

Damage to the intervertebral discs (IVDs) occurs due to aging or excessive mechanical stress, causing a series of IVD‐related degenerative diseases, such as spinal disc herniation and spondylosis. These IVD‐related diseases are difficult to cure, partially because the regeneration ability of IVDs is not sufficient. As a novel strategy for treatment of IVD‐related diseases, mesenchymal stem cell transplantation to the damaged discs has been reported in animal studies. To further develop and improve this approach, it is necessary to gain a better understanding of the molecular network regulating IVD development by critical transcription factors. Recent findings reveal that during IVD development, nucleus pulposus and annuls fibrosus differentiation is coordinated by a series of transcription factors, such as *Mkx*, *Pax1*, *9*, *Shh*, *Foxa1, 2*, *T‐Brachyury*, and *Sox5, 6, 9*. The combination of mesenchymal stem cell transplantation with the regulation of these molecules may provide a novel strategy for treatment of degenerative disc diseases.

## Graphical abstract

To develop an innovative regenerative therapy for IVD‐related diseases, knowledge of the molecular network in IVD development and homeostasis should be useful. In particular, identification of specific transcription factors regulating IVD development is key to uncover the gene expression network. These critical transcription factors could be targets or tools to develop regenerative medicine for IVD‐related diseases. In this review, we summarize the recent progress on the analysis of the critical transcription factors in IVD development and repair.

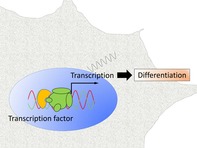

## 1 INTRODUCTION

**Figure 1:**
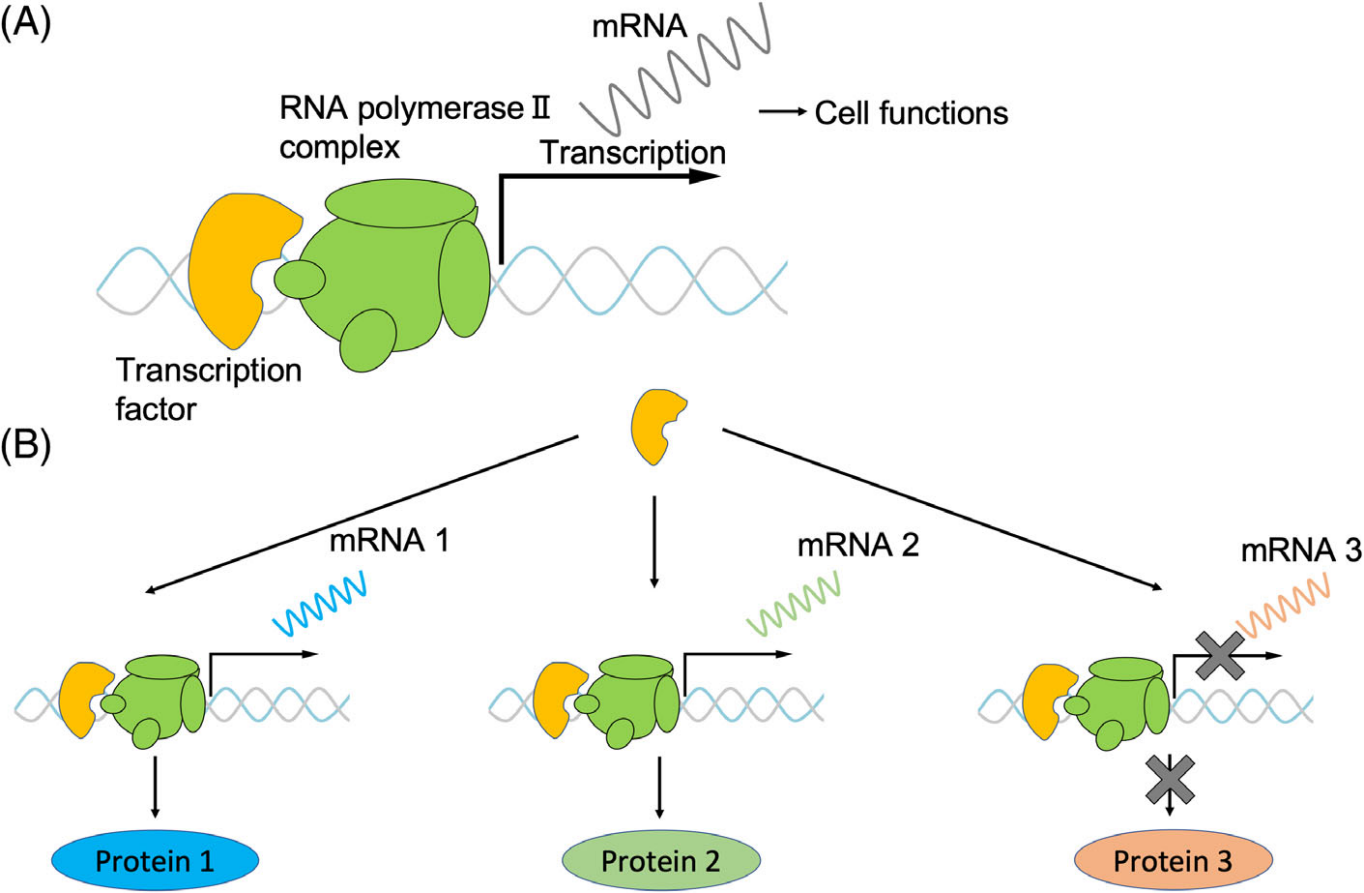
Shema of a function of a transcription factor. A, Typically, transcription factors bind the promotor region and adjust the expression of downstream genes. B, Transcription factors adjust expressions of multiple genes and have a function to differentiate into mature cells

Intervertebral discs (IVDs) are fibrocartilaginous structures connecting adjacent vertebrae in the spinal column.^1^ Degeneration or damage of IVDs due to aging or excessive mechanical loading could result in lumbar spine diseases, such as intervertebral disc herniation, spinal spondylosis, and spinal canal stenosis.^2^, ^3^ Patients with these diseases suffer from severe pain, which limits their productivity and daily activities. IVD is among the largest avascular tissues in the body and has poor self‐healing potential,^4^ which makes damage to IVDs irreversible, leading to degenerative spondylosis. There are only a few therapeutic approaches available to IVD‐related diseases. In many cases, therapies for IVDs related diseases may be limited to relieving pain. Surgical approaches, such as discectomy of IVD herniation, also provide relief from severe pain; however, this operation does not affect the progression of the diseases.^4^ To develop an innovative regenerative therapy for IVD‐related diseases, knowledge of the molecular network in IVD development and homeostasis should be useful. In particular, identification of specific transcription factors regulating IVD development is key to uncover the gene expression network (Figure 1). These critical transcription factors could be targets or tools to develop regenerative medicine for IVD‐related diseases. Toward this end, we summarize the recent progress on the analysis of the critical transcription factors in IVD development and repair.

## 2 THE STRUCTURAL ANALYSIS AND DEVELOPMENTAL PROGRAM OF IVDs

**Figure 2:**
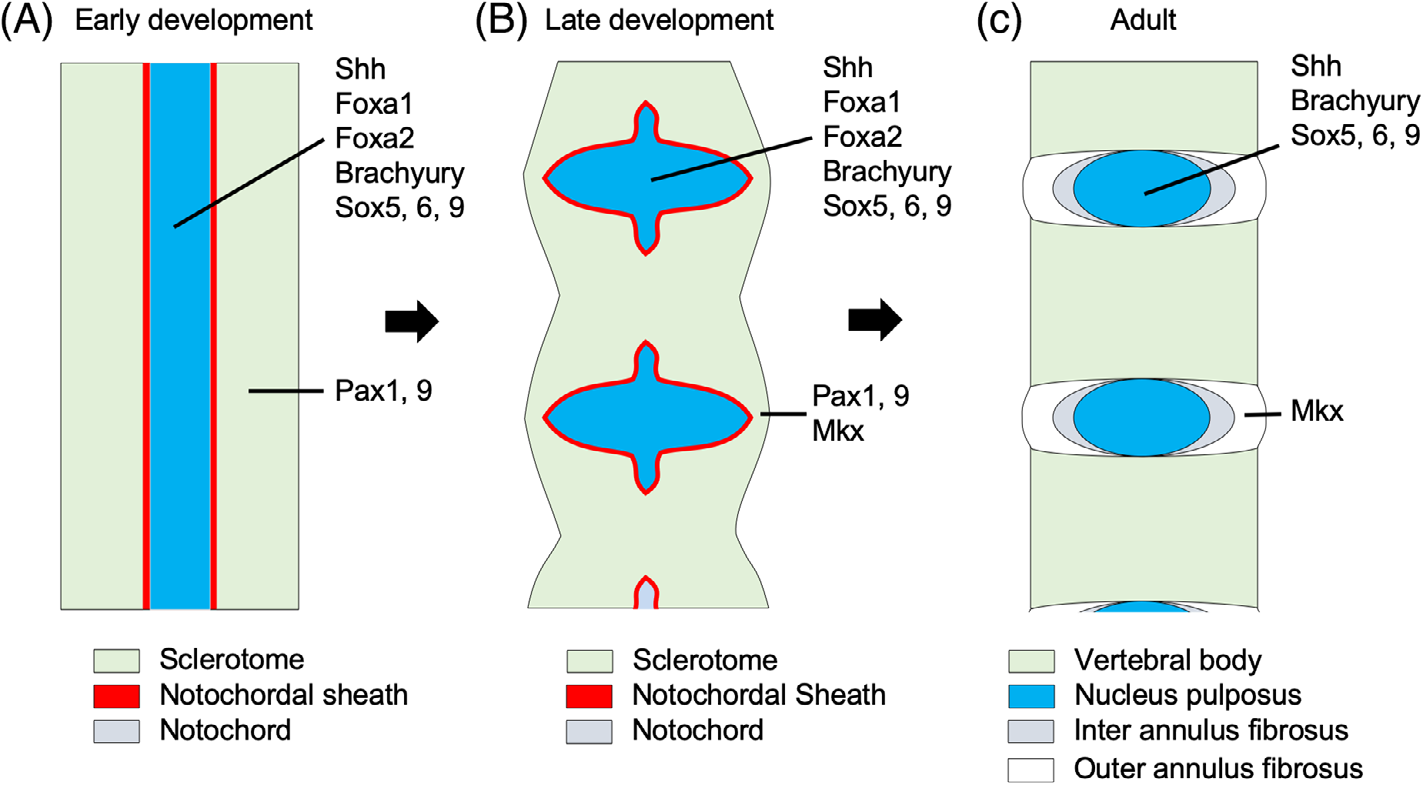
Schema of development of the intervertebral disc. A, Sclerotome cells condense around the notochord. B, Sclerotome cells form a metameric pattern of more‐condensed regions, which become vertebral bodies, and less‐condensed regions, which form the AF. The notochord expands within the future IVD to form the NP. C, Basic structure of the intervertebral disc. The AF cells form a lamellar structure

IVDs consist of three major components: the nucleus pulposus (NP), the annulus fibrosus (AF), and the cartilaginous endplate (EP). NP is a jelly‐like material located in the center of an IVD. The main function of NP is to stabilize the IVD against mechanical stress.^5^ AF is the fibrous tissue surrounding the NP. AF is composed of the outer AF (OAF) and the inner AF (IAF). The OAF is a highly organized collagenous structure consisting mainly of type I collagen, whereas the IAF contains organized type I and type II collagen.^5^, ^6^ EP comprises cartilaginous tissues between the IAF and the NP. Mechanical pressure to the IVDs is well absorbed and balanced into the NP and the AF structure. Capillary vessels in the EP provide nutrition to the entire IVD.^6^ The developmental origins of these components are different. Based on cell fate tracing analysis in mice,^8^ the notochord is the origin of the NP and the sclerotome is the origin of the AF, EP, and vertebral body. At E10.0, a notochord sheath is formed around the notochord and vertebral body formation begins. At E12.5, sclerotome cells migrate and condense around the notochord (Figure 2A).^8^ In a somite pattern, the dense part of the sclerotic cells forms the vertebral body and the sparse part becomes the annulus. The notochord region that expands within the future IVD forms the NP (Figure 2B).^9^ Notochord cells dominantly produce glycosaminoglycan‐rich extracellular matrix‐like aggrecan. Sclerotome cells in less‐condensed regions produce collagen‐rich extracellular matrix‐like type I and type II collagen. In this way, the formation of the vertebral body, endplate, nucleus pulposus, and annulus fibrosus is completed to form the adult IVD (Figure 2C). The origin of development differs for each tissue constituting the intervertebral disc, and therefore transcription factors important for development vary depending on each constituent tissue. Since the embryonic origin of each component is different from one another, the transcription factors involved in each specific cell type differentiation are different.

## 3 TRANSCRIPTION FACTORS RELATED TO NP DEVELOPMENT

*Sonic Hedgehog* (*Shh*) is specifically expressed in the notochord at E9.5 in mice and plays an important role in the formation and maintenance of the notochord.^10^ *Smoothened* (*Smo*) functions to activate *Shh* signaling. It was reported that mice with conditional knockouts of *Smo* and *Shh* do not form a notochord sheath and therefore, cannot form the NP.^10^ A recent report revealed that the expression of *Shh* at E12.5 is notably higher than that at P0 with notochord‐derived cells. It indicates *Shh* functions mainly in the developmental phase.^11^ In addition, *Shh* signaling activates Wnt (a portmanteau of Wingless and Int‐1) signaling and increases *T‐Brachyury* and *Aggrecan* expression within adult NP (Table 1).^12^ Thus, *Shh* is considered to be an important transcription factor not only in NP development but also in NP maintenance.

**Table 1:** Summary of transcription factors related to NP and AF developments

Name	Expression site	Onset of expression (mice)	Development	Homeostasis
Major transcription factors for NP development				
Shh	Notochordal cells	E9.5‐	Formation of notochordal sheath and the NP^10^	Regulation of the expression of Brachyury and Aggrecan^12^
Foxa1, Foxa2	Notochordal cells	E8.5‐	Adjustment of the expression of Shh^13^	unknown
T‐Brachyury	Notochordal cells	E10.5‐	Formation of notochord^13^, ^14^	Regulation of the expression of FGF8 and Axin2^15^, ^16^
Sox5,6,9	Notochordal and sclerotome cells	Sox5,6: E11.5‐	Formation of the NP and the IAF^19^, ^20^	Regulation of the expression of Col2 and Aggrecan^18^
		Sox9: R10.5‐		
Pax1, 9	Sclerotome cells	E10.5‐	Regulation of the cartilage related genes^26^	Unknown
	(early stage)			
Major transcription factors for AF development				
Name	Expression site	Onset of expression (mice)	Development	Homeostasis
Pax1, Pax9	The OAF cells	E10.5‐	Unknown	Unknown
Mkx	The OAF cells	E14.5‐	Formation of the OAF^24^	Maintenance of the OAF homeostasis?^24^

*Note*: Multiple transcription factors work in both the developmental and the postnatal stage.

*Foxa1* and *Foxa2* are expressed in the notochord at E8.5 in mice (Figure 2A). *Foxa1* single knockout mice have severely deformed IVDs and *Foxa2* single knockout is fatal in early development, and hence notochord formation cannot be analyzed in these mice. However, in *Shh‐CreERT: Foxa2/Foxa1* knock out mice, notochord formation is disturbed.^13^ These results indicate the possibility of functional redundancy between *Foxa1* and *Foxa2* and suggest that these transcription factors together are essential in notochord formation. These transcription factors are involved in the expression of *Shh* in the notochord (Table 1).^13^ To date, the expression pattern of *Foxa1* and *Foxa2* in the adult phase is not reported yet and it is of interest to test the potential functions of these genes in the adult.

*T‐Brachyury* is a transcription factor that plays a critical role in embryonic mesoderm development, particularly for the formation of the notochord (Figure 2A) (Table 1).^14^ Recent studies have shown that *T‐Brachyury* is expressed and functions in the notochord at E10.5. ^15^ Shh Cre inducible T‐Brachyury shRNA expressing *T‐Brachyury* knockdown mice show a phenotype with a costal level of vertebral malformation or loss because of the loss of the notochordal cells, which indicates that *T‐Brachyury* is essential for normal spine formation.^15^ Even after the completion of the NP formation, *T‐Brachyury* is still strongly expressed in the NP cells, particularly in the notochordal cells. It is also reported that *T‐Brachyury* regulates the expression of fibroblast growth factors 8 and Axin 2, which are related to disc degeneration.^16; 17^ Thus, *T‐Brachyury* has a function in not only NP development but also NP maintenance (Table 1).

*Sox5*, *6*, and *9* are related to the formation of NP and IAF. *Sox9* knock out mice show complete absence of cartilage; therefore, *Sox9* is considered as a master transcription factor in chondrogenesis.^18^ *Sox5* and *Sox6* are also known to act as transcription factors in chondrogenesis by enhancing the function of *Sox9*.^19^ In view of IVD development, *Sox5* and *Sox6* are expressed in the sclerotome and the notochord at E11.5 in mice (Figure 2A).^20^ They regulate the expression of type II collagen and aggrecan and are involved in the formation of the vertebral body and the IAF. In *Sox5/Sox6* double‐knockout mice, the formation of the vertebral body and the IAF was impaired, resulting in the inhibition of NP formation.^20^ Furthermore, *Sox9* was also expressed in the sclerotome and the notochord at E10.5 in mice and was involved in the formation of the NP, IAF, and vertebral body (Table 1).^21^

## 4 THE TRANSCRIPTION FACTORS RELATED TO AF DEVELOPMENT

Whereas there are substantial reports to elucidate the NP development, there are few reports that explore AF development. AF consists of two different types of tissues, the IAF and the OAF, and the molecular network for each tissue's development should be determined. *Pax 1* and *Pax 9* play a significant role in the development of IVDs and are strongly expressed in the sclerotome at E10.5 in mice (Figure 2A). The expression of these genes is under control of Shh from the notochord.^22^ *Pax1* is expressed throughout sclerosis in the early stage of development, but its expression gradually decreases in the vertebral body part, and in the later stage of development, expression is limited to the AF, especially the OAF. ^23; 24^ *Pax1* knockout mice have vertebral and intervertebral disc dysplasia and rib dysplasia, and the notochord cannot form the NP structure even in the late stage of development.^23^ Recently, it was reported that *Pax1* functions mainly in the IAF in the early‐to‐middle developmental stage and showed *Pax1*‐mediated signaling to the notochord and its role in the regulation of cell proliferation.^25^ Another report showed that *Pax1* and *Pax9* regulated the expression of the cartilage‐related genes known to be regulated by *Sox5*, *Sox6*, and *Sox9* in the early developmental stage.^26^ Moreover, *Pax1* and *Pax9* are downregulated by way of a negative feedback mechanism through *Sox5*, *Sox6*, and *Sox9* expression (Table 1).^26^ Thus, *Pax1* and *Pax9* function mainly for the IAF development as separation of the IAF and the OAF. However, it is still unknown why the expression of *Pax1* and *Pax9* is kept in the OAF with warranting further analysis.

Recently, *Mohawk* (*Mkx*) has been reported as an essential transcription factor for OAF development.^27^ *Mkx* is a member of the three‐amino‐acid loop superclass of atypical homeobox genes belonging to the Iroquois family.^28^ The expression of *Mkx* in the syndetome is detectable at E12.5 and its expression is maintained even in matured ligament cells.^29^ In IVD, *Mkx* is mainly expressed in the OAF at the early developmental phase until well after maturity (Figure 2B).^27^ In *Mkx* knockout mice, the AF was found to be thinner than that in the wild‐type mice, and it was also confirmed using electron microscopy that the diameter of collagen fibrils had reduced in *Mkx* knockout mice.^27^ Moreover, in the OAF cells, multiple genes associated with ligament tissue synthesis were downregulated in *Mkx* knockout mice.^27^ Taken together, these phenotypes show that *Mkx* plays an essential role in OAF formation (Table 1).

*Mkx* is known as a transcription factor that has an essential role in tendon and ligament development. *Mkx* knockout mice show a reduced tendon mass but no decrease in the number of tendon cells (the same phenotype was seen in the AF).^26^, ^28^ In *Mkx* knockout rats, the heterotopic ossification of the tendon has occurred via failed tenogenesis.^30^ Furthermore, some reports focused on the function of *Mkx* after tendon and ligament maturation, and they showed that the reduction of *Mkx* expression induces ligament degeneration, and that appropriate mechanical stress, applied via *Mkx* expression in vitro and in vivo, was essential for tendon homeostasis.^31^ From these reports, it can be predicted that *Mkx* has an essential function not only in tendon maturation but also in the maintenance of tendon homeostasis. In the OAF, the expression of Mkx is also kept after its maturation^24^; thus, we can hypothesize that Mkx plays a role in maintaining OAF homeostasis, and further analysis is expected in the future.

As essential transcription factors for tendon and ligament development, *Scleraxis* (*Scx*) and *Egr1* have also been studied extensively. *Scx* is a helix‐loop‐helix (bHLH) transcription factor that is expressed in tendon progenitors.^31^, ^32^ *Scx* is also expressed in the AF during the developmental phase.^33^ The OAF and ligaments are both fibrous tissues that consist of mainly type I collagen and both perform the same function, that is, to connect bone to bone and contribute to the stability between them. Therefore, we can predict that these tissues are similar in view of development. Interestingly, in *Scx* knockout mice, significant hypoplasia of the tendon is seen whereas the IVDs structure is normal. This difference may indicate an underlying property that differentiates the OAF and ligaments from tendons. *Egr1* is a member of the *Egr* family of C2H2‐type zinc finger transcription factors.^34^ *Egr1* and *Egr2* are expressed in the developing tendon and play important roles in tendon formation.^35^ Unfortunately, the function of *Egr1* in the development of IVDs is not well defined. In *Egr1* knockout mice, the tendon was found to be hypoplastic and the expression of *Scx* was impaired, whereas the expression of *Mkx* was maintained. Therefore, it can be predicted that *Mkx* and *Egr1* have different pathways for tendon development.^36^ In the future, it may be attractive to explore this aspect by focusing on the function of Egr1 toward the AF.

## 5 STEM CELL THERAPY INDUCED BY TRANSCRIPTION FACTORS

To develop regenerative therapy for treating IVD damage, a number of cell transplantation studies into IVDs have been reported. These studies can be divided into three categories: cell induction using growth factors, cell transplantation using mature cells, and cell transplantation using stem or progenitor cells. Among them, mesenchymal stem cell (MSC) transplantation for NP regeneration has been well‐developed by many groups.^36^, ^37^, ^38^, ^39^, ^40^ The clinical studies of MSC transplantation in NP have already been conducted and have yielded successful results to some extent.^41^, ^42^, ^43^, ^44^, ^45^ However, whether transplanted MSCs could successfully differentiate to NP cells to reconstruct IVDs remains unclear. In this regard, identification or induction of more tissue‐specific progenitor cells for IVDs may improve the therapy. One study reported that TIE 2 and GD 2 are markers of IVD progenitor cells.^47^ Another study attempted to induce the formation of notochordal cells from iPS cells.^48^ These cells could be applied to stem cell therapy for NP in the near future.

Regarding the reconstruction of the damaged OAF, the transplantation of OAF cells or MSCs into IVD injury sites has been reported in animal models.^48^, ^49^, ^50^ However, the transplanted OAF cells did not maintain the OAF cell characteristics and were not able to synthesize sufficient collagen fibers.^50^ This would be partly because the differentiation of MSCs to the OAF is not well‐directed in the transplantation region.^48^, ^50^ To overcome this issue, MSCs modified by IVD‐specific transcription factor expression could be applied. MSCs that overexpress *Mkx* acquire the ability to produce multiple tendon‐ and ligament‐associated proteins and synthesize ligament‐like tissues.^23^, ^51^, ^52^ Based on these findings, the transplantation of these cells into the IVD injury in the animal model results in ligament‐like tissue synthesis that has sufficient physical properties.^24^ This successful model builds a case for understanding the function of a transcription factor in tissue development and using it as a therapeutic tool.

There are also reports on methods of inducing MSCs to NP cells using growth factors.^53^, ^54^ One study utilized the pellet culture of human mesenchymal stem cells and human adipocyte‐derived stem cells with GDF6, and successfully induced the expression of *Sox9* and *T‐Brachyury*.^56^ As for *Mkx*, a marker of the annulus, there was a report that the expression of *Mkx* was induced when TGFB was added to mouse tendon‐derived cells.^57^ There are still few reports on stem cell induction using growth factors for IVDs, and further research is expected in the future.

## 6 CONCLUSION AND FUTURE DIRECTION

**Figure 3:**
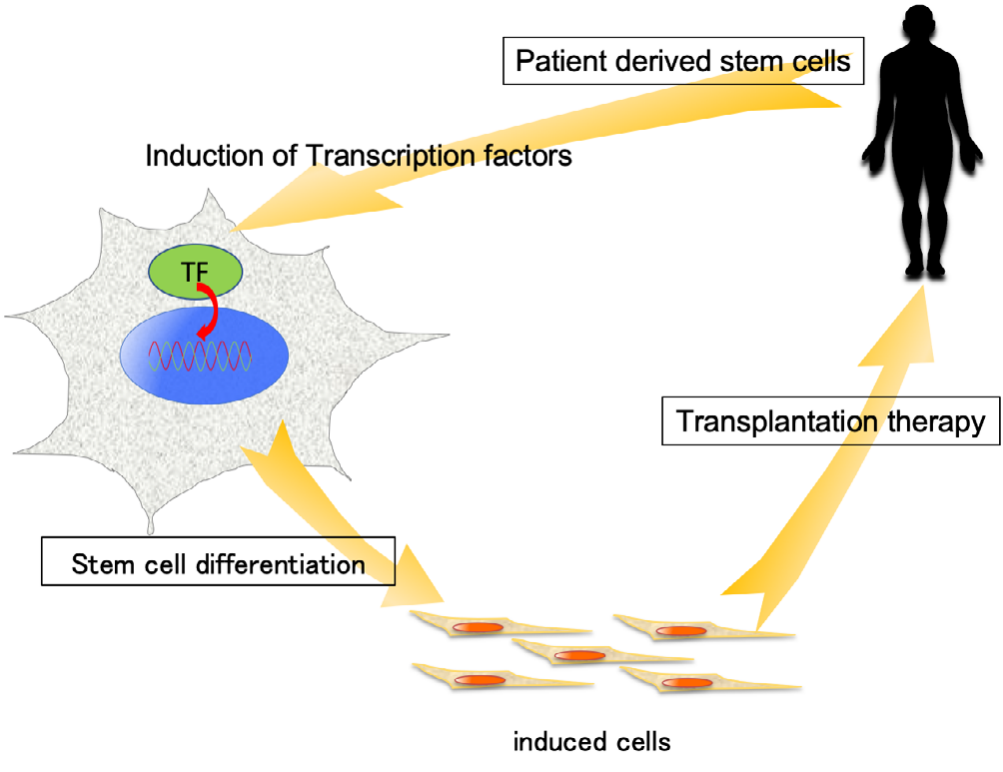
Schema of stem cell‐based regenerative therapy. A more sophisticated method for induction of differentiation is needed

The developmental mechanism of the IVD has recently been uncovered with identifications of critical transcription factors in IVD development. In the next stage, it is essential to reveal the transcriptional network coordinated by these transcription factors during IVD development. For this purpose, chromatin immunoprecipitation and/or single cell analysis should be performed. As for clinical application, cell therapy or chemical compounds targeting these transcription factors could be tested to repair IVDs (Figure 3).

## CONFLICT OF INTEREST

The authors declare no conflicts of interests.

## AUTHOR CONTRIBUTIONS

R. N. and H. A. wrote the paper.
